# Disentangling the Causal Effects of Education and Participation Bias on Alzheimer Disease Using Mendelian Randomization

**DOI:** 10.1212/NXG.0000000000200307

**Published:** 2025-11-07

**Authors:** Aadrita Chatterjee, Clémence Cavaillès, Neil M. Davies, Kristine Yaffe, Shea J. Andrews

**Affiliations:** 1 Department of Psychiatry and Behavioral Sciences, University of California, San Francisco;; 2 San Francisco Veterans Affairs Health Care System, CA;; 3 Division of Psychiatry, University College London, United Kingdom;; 4 Department of Statistical Science, University College London, United Kingdom; and; 5 K.G. Jebsen Center for Genetic Epidemiology, Department of Public Health and Nursing, Norwegian University of Science and Technology, Hogskoleringen, Norway.

## Abstract

**Background and Objectives:**

People with university degrees have a lower incidence of Alzheimer disease (AD). However, the relationship between education and AD could be due to selection, collider, or ascertainment biases, such as lower familiarity with cognitive testing or the fact that those with degrees are more likely to participate in research. In this study, we use 2-sample Mendelian randomization (MR) to investigate the causal relationships between education, participation, and AD.

**Methods:**

We used genome-wide association study summary statistics for educational attainment, 3 measures of participation, AD (clinically diagnosed AD), and AD/ADRD (AD and related dementia; includes clinical diagnosis and family history). Independent genome-wide significant single-nucleotide variations (SNVs) were extracted from the exposure data sets and harmonized with the outcome SNPs. Fixed-effects inverse variance–weighted meta-analysis was the primary MR method; Cochran Q statistic and MR Egger intercept were used to test for heterogeneity and pleiotropy, and radial MR was used to identify outliers. Sensitivity analyses included MR Egger, weighted median, and mode. Bidirectional analyses were used to test whether AD pathology affects participation, and multivariable MR (MVMR) assessed independent exposure-outcome effects.

**Results:**

Educational attainment reduced the risk of AD (OR_IVW_ 95% CI 0.70 [0.63–0.79], *p* = 8e-10), and the results were robust based on sensitivity analyses. However, education increased the risk of AD/ADRD, although the results were not robust in sensitivity analyses (OR_IVW_ 95% CI 1.09 [1.02–1.15], *p* = 0.006). Participation in the mental health questionnaire reduced the odds of AD (OR_IVW_ 95% CI 0.325 [0.128–0.326], *p* = 0.01). When adjusting for participation in MVMR, education remained associated with a reduced risk of AD (OR_IVW_ 95% CI 0.76 [0.62–0.92], *p* = 0.006).

**Discussion:**

Univariable MR analyses indicated that education and participation reduced the risk of AD. However, MR also suggested that education increased the risk of AD/ADRD, highlighting the inconsistencies between clinical and proxy diagnoses of AD, as proxy-AD may be affected by selection, collider, or ascertainment bias. MVMR suggested that education remained associated with reduced AD risk after adjusting for participation and the protective effect of education may be due to other biological mechanisms, such as cognitive reserve.

## Introduction

Epidemiologic studies have found that people with higher educational attainment have a lower incidence of Alzheimer disease (AD). These associations may be due to cognitive reserve, whereby education reduces the impact of neuropathologic lesions in the brain.^1^ Increased educational opportunities and improved cardiovascular health have also been linked to the observed secular decline in the incidence of dementia.^2^ However, the observed protective effect of education on AD may be influenced by participation bias because individuals with more education are more likely to participate in research studies.^3^ In a study examining participation in clinical research and education, individuals with higher levels of education, such as college students, exhibited a greater likelihood of participation in clinical trials.^4^ When comparing participants from the National Alzheimer's Coordinating Center sample (NACC) AD Research Centers to those from the nationally representative Health and Retirement Study (HRS), NACC participants had higher levels of education compared with HRS participants, further supporting a positive relationship with education and participation in clinical studies.^5^

**Figure 1:**
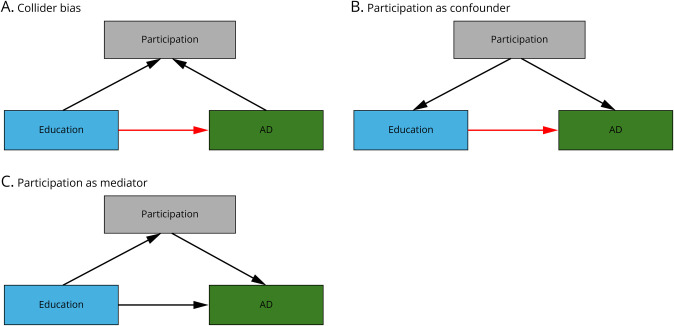
Directed Acyclic Graphs Illustrating Different Relationships Between Our Traits of Interest Directed acyclic graph (DAG) illustrating (A) collider bias: both education and AD genetic risk influence participation, creating spurious education-AD association in the population; (B) confounder bias: participation affects both education and AD risk, confounding the inferred association between education and AD risk; and (C) model of participation mediating relationship between education and AD risk. The black arrows represent causal effects, and the red arrows represent distorted associations. Note that multivariable Mendelian randomization (MVMR) can only assess the mediation relationships depicted. AD = Alzheimer disease.

Selection and collider biases can influence the observed relationship between education and AD. Selection bias occurs when the participants in a study are a nonrandom sample of the general population.^6^ One source of selection bias is differences in the likelihood of participation across the population (e.g., more educated people are more likely to participate in research studies). These differences in participation rates can lead to selection bias and lack of representativeness in ascertained samples (e.g., they may be healthier and have lower rates of disease). A more subtle consequence of differential participation is collider bias, which can be induced if both the exposure (education) and outcome (genetic liability to AD) are conditioned on, either by being selected into a sample or statistically (e.g., if both educational attainment and genetic liability to Alzheimer affect participation). Collider bias can induce spurious correlations between the exposure and outcome, which are not present in the population and do not indicate a causal effect of the exposure on the outcome^6^ (Figure 1A). For example, selective participation could create a spurious association between higher education and lower prevalence of AD within the study sample. This bias occurs not because education directly influences AD risk, but because education level and genetic liability to AD affect the likelihood of ascertainment into an analytic sample. Participation can also be a confounder because it can affect education and AD risk (Figure 1B). However, in this study, we will test whether participation could mediate the direct causal effect of education on AD to determine whether participation bias explains this relationship (Figure 1C).

The observed effects of education in Mendelian randomization (MR) studies could be due to either the causal effects of education or participation bias. This is important because if the effects are due to collider bias, then educational attainment is unlikely to be an effective modifiable risk factor of AD, and recent secular changes in educational attainment are unlikely to affect rates of disease in the population.

MR uses instrumental variables—genetic variants associated with the exposure of interest—to investigate the causal effect of an exposure on an outcome.^7^ Owing to the random inheritance of alleles at conception and meiosis, conditional on parental genotype, genetic variants associated with the exposure are less confounded than observed measures.^8^ Furthermore, reverse causation is impossible because the environment cannot affect germline genetic variation after conception. Multivariable MR (MVMR) extends this approach using genetic variants associated with multiple exposures as instruments to estimate the “direct” and indirect effects of each exposure on the outcome.^8^ MVMR analyses have shown a bidirectional effect between education and cognitive ability; when examining the total effects of education on AD, it was found that cognitive abilities mediate the impact of education on AD.^9^ In addition, cortical surface area, volume, and intrinsic curvature were found to be associated with educational attainment.^10^

The effects of education on AD and participation in an optional mental health questionnaire (MHQ) on AD have been established using MR^9,11^ However, further research is needed to determine whether participation bias affects the observed relationship between education and AD. This requires incorporating additional measures of both participation and AD, including genome-wide association studies (GWASs) of clinical AD and GWAS by proxy (GWAX), which considers family history of dementia. Notably, the weighted participation measure, which uses inverse probability weighting (IPW), can mitigate some issues related to collider and selection biases by making the sample more representative of the broader population.^12,13^ A simulation study also found that the impact of selection bias is generally considered less significant compared with biases such as pleiotropy or population stratification in MR studies.^14^ In addition, MVMR allows for the estimation of the independent effects of both education and participation on AD risk to assess whether the protective effect of education on AD is mediated by participation or whether it operates through an independent pathway.^15^ This study used genetic correlations and bidirectional and multivariable two-sample MR to investigate the causal relationships between education, participation, and AD and determine whether education's effect on AD is due to participation bias.

## Methods

To address our research question, whether the effect of education on AD is mediated by participation, we designed our study to test specific hypotheses using MR. First, we examined genetic correlations to assess whether a shared genetic architecture exists between education, participation, and AD traits, providing the rationale for our analyses. Next, we used forward MR to test the hypothesis that education and participation caused AD, using 3 distinct measures of participation, each with systematic biases due to differences in how participation was assessed. We tested whether participation and education are caused by AD to assess reverse causation. Finally, we conducted MVMR to evaluate the alternate hypothesis: that the effect of education on AD was mediated by participation. An overview of the study design is demonstrated in eFigure 1.

### Data Sources

GWAS summary statistics were obtained for each exposure and outcome data set (eTable 1). The GWAS for education measured educational attainment as the number of years of schooling (n = 3,037,499, n_loci_ = 3,952).^16^ For participation, we used a primary measure involving completion of an optional MHQ of the UK Biobank (n = 451,036, n_loci_ = 32) because it was the most statistically powered.^11^ Two other GWASs of participation were used to validate our results across different measures, and we included a weighted GWAS based on a probability model that has individual participation probabilities as the outcome (n_effective_ = 102,215, n_loci_ = 28).^13^ The probability model corrected for nonresponse bias by giving greater weight to overrepresented and underrepresented individuals, thus creating a more representative pseudo-population that mimics the Health Survey England, which was used as the reference sample. The next measure of participation used estimated factor scores for the general “I don't know” behavior across UK Biobank survey questions (n = 360,628, n_loci_ = 35).^17^ Two GWASs were used for AD. The first involved clinically diagnosed AD cases (n = 94,437, n_loci_ = 25 LOAD risk loci), which we refer to as “AD.”^18^ The second leveraged clinical case-control series, in addition to self-reported family history of dementia, to conduct a GWAX (n = 788,989, n_loci_ = 72) of AD and related dementias, which we refer to as “AD/ADRD.”^19^ All cohorts included age and sex as covariates, and all individuals were of European ancestry. All GWASs were standardized using “MungeSumstats” version 1.10.1.^20^

### Genetic Correlations

We estimated genetic correlations between each trait using linkage disequilibrium score regression implemented by GenomicSEM v0.0.5.^21^ Genetic correlations quantify the degree of shared genetic influence between 2 traits, representing the proportion of variance in these traits that can be attributed to common genetic influences and can be described as strong (rg > 0.6), medium (rg 0.2–0.6), or weak (rg < 0.2).^22^ Shared genetic architecture can result from horizontal or vertical pleiotropy, confounding, or selection bias. Horizontal pleiotropy occurs when a genetic variant influences the outcome through pathways other than the exposure, violating a core assumption of MR (e.g., if a variant directly affects AD risk independently of education).^8^ MR tests for vertical pleiotropy whereby the genetic variants influence the outcome only through the exposure (e.g., if a variant affects AD risk solely via its effect on education).^8^ This allows us to determine whether the shared genetic architecture influences both traits through independent pathways or whether the effect on one trait is mediated by its influence on the other. LDSC quantifies heritability by assessing the correlation between genetic variants across the genome and the trait of interest, using a European reference panel from 1,000 Genomes to estimate linkage disequilibrium (LD).^22^

### MR

#### Selection of Genetic Instruments and Data Harmonization

We first performed clumping (*r*^2^ = 0.001, 10-Mb clumping window, EUR reference) to identify and retain independent genome-wide significant (*p* < 5e-8) single-nucleotide polymorphisms (SNPs) using the OpenGWAS API.

For instrumental variables missing from the outcome GWAS data set, LD proxies were identified using LDlinkR version 5 (reference = EUR; *r*^2^ > 0.8).^23^ The exposure and outcome data sets were harmonized to ensure that their SNP effects corresponded to the same effect allele, with palindromic variants inferred using their allele frequencies.^24,25^ The *APOE* region (19:44912079-19:45912079, build 37) was removed because of its known pleiotropic effects.^26^

#### Statistical Analysis

MR is a statistical technique that leverages genetic variants as instrumental variables to estimate causal relationships between an exposure and an outcome. MR relies on the principle that genetic variants are randomly assigned at conception and thus free from confounding, making them ideal instruments for causal inference. MR holds 3 key assumptions: the genetic variants associate with the exposure; there are no controlled confounders of the genetic variant-outcome association; all the effects of the genetic variant on the outcome are mediated via the exposure of interest.^8^

We performed univariable MR to estimate the causal effect of education on each participation measure and AD. We also estimated the effect of genetic liability to AD on education and each participation measure. Fixed-effects inverse variance–weighted (IVW) analysis was the primary analysis method as it is the most precise. The fixed-effects IVW method weighs variant-exposure and variant-outcome associations by the inverse of their variances to provide a single causal estimate, assuming that all instruments are valid and there is no unbalanced horizontal pleiotropy.^27^

#### Diagnostics

To test the validity of the instrumental variable assumptions, we used F-statistics to assess the strength of the genetic instruments, the Cochran Q test for heterogeneity, and the MR Egger regression intercept for horizontal pleiotropy.^8^ Higher F-statistics (F-statistic >10) indicate stronger instruments and are less likely to lead to weak instrument bias.^8^ Radial MR version 1.0 was used to detect outliers that were omitted from the MR analysis.^28^

#### Sensitivity Analyses

For each MR analysis, sensitivity analyses were performed to test the robustness of the causal association between the exposure and outcome, addressing either heterogeneity or horizontal pleiotropy. These include random-effects IVW, MR Egger, weighted median (WME), and weighted mode–based estimator (WMBE), with each method having different assumptions.^29-31^ The assumption of no unbalanced horizontal pleiotropy is relaxed in MR Egger; WME combines multiple genetic instruments to estimate causal effects and provides valid results when at least 50% of the instruments are valid; WMBE is unbiased when the modal estimate across the SNPs is from a valid (i.e., nonpleiotropic) SNP.^8^ We interpreted our results as robust evidence of a causal effect when the IVW analysis was significant (*p* < 0.05) after outlier removal and where there was no evidence of heterogeneity (*p* > 0.05) or pleiotropy (*p* > 0.05). In the presence of heterogeneity or pleiotropy, causal associations were deemed robust if at least one of the sensitivity analyses was also significant (*p* > 0.05) and had the same direction of effect.

#### Multivariable MR

Multivariable MR is an extension of univariable MR that incorporates instrumental variables associated with multiple exposures in the analysis. Therefore, MVMR allows us to evaluate the direct causal effect of an exposure on an outcome while univariable MR only estimates the total causal effect.^8^ MVMR can be used to overcome the strong assumptions required for causal inference in traditional non-IV mediation methods such as no measurement error in the mediator or exposure.^15^ In MVMR, SNPs from the 2 exposure data sets were combined and clumped (*r*^2^ = 0.001, 10-Mb clumping window, EUR reference), and independent genome-wide significant SNPs (*p* < 5e-8) were extracted from each exposure data set. Proxy variants were identified for any SNPs not present in either of the exposure data sets. Exposure SNPs were then extracted from the outcome GWAS, and proxy SNPs for the outcome were identified for variants not present in the outcome GWAS. The exposure and outcome data sets were harmonized using TwoSampleMR version 0.5.11. We performed MVMR and evaluated the results using diagnostics and sensitivity analyses to determine whether the effect of education on AD was being mediated by participation bias, using the MVMR package version 0.3 and MendelianRandomization package version 0.9.0.^32,33^ A robust causal effect in the MVMR analysis was defined similarly to the univariable analysis.

### Standard Protocol Approvals, Registrations, and Participant Consents

Ethical approval was granted in the original studies. All participants provided written informed consent for participation in the original studies.

### Data Availability

All statistical analyses were performed using R version 4.3.0. The code used to conduct the analyses is available at github.com/AndrewsLabUCSF/Aadrita-AD-participation-education. Original summary statistics can be found at the following websites: niagads.org/datasets/ng00075, ebi.ac.uk/gwas/publications/35379992, thessgac.org/data, ebi.ac.uk/gwas/publications/33563987, ebi.ac.uk/gwas/publications/37106081, and ebi.ac.uk/gwas/publications/37386106.

## Results

### Genetic Correlations

**Figure 2:**
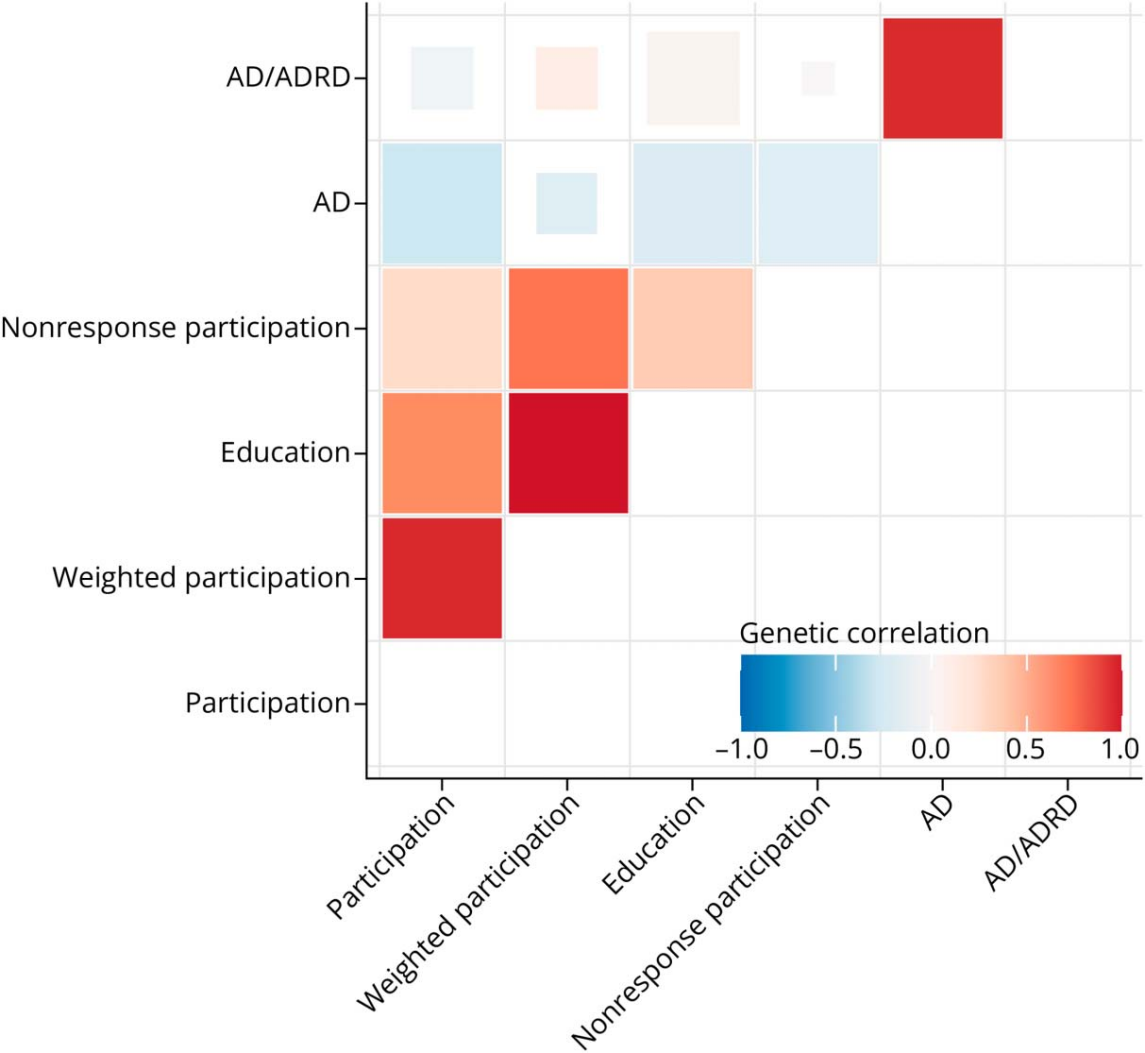
Genetic Correlations of Education, Participation, Weighted Participation, Nonresponse Participation, and AD and AD/ADRD Genetic correlations between 3 different participation measures, education, AD and AD/ADRD. Blue indicates a negative correlation while red indicates a positive correlation. *p* values are represented by the size of the colored squares where larger squares represent a smaller *p* value. Genetic correlations were calculated using GWAS summary statistics from previous studies and LD score regression. Nonresponse participation is reverse coded. AD = Alzheimer disease; GWAS = genome-wide association study; LD = linkage disequilibrium;

We found medium and strong positive genetic correlations between the 3 participation measures (Figure 2). Education exhibited the strongest positive correlation with weighted participation, followed by participation and nonresponse participation. Education was also negatively associated with AD. However, it was not correlate with AD/ADRD. By contrast, participation was weakly negatively correlated with both AD and AD/ADRD (Figure 2).

### Higher Educational Attainment Is Associated With a Reduced Risk of AD, but an Increased Risk of AD/ADRD

**Figure 3:**
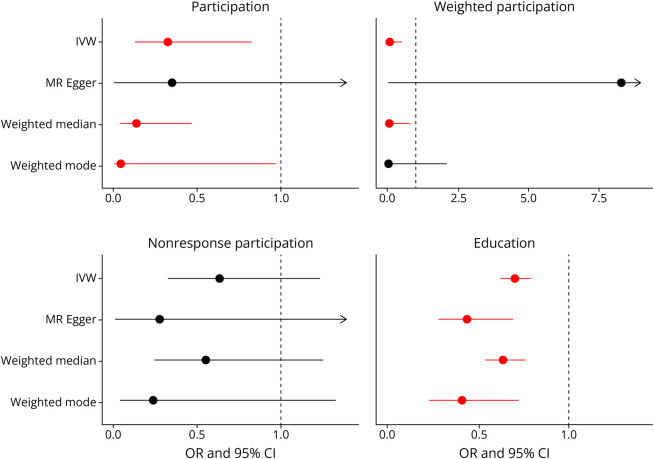
Univariable Odds Ratio of the Association Between Participation Measures, Education, and AD Forest plots showing the MR odds ratio for the 4 different traits (participation, weighted participation, nonresponse participation, and education) and AD based on the univariable analyses. Red represents significant results, and arrows represent CI intervals that continue past the x-axis. AD = Alzheimer disease.

**Figure 4:**
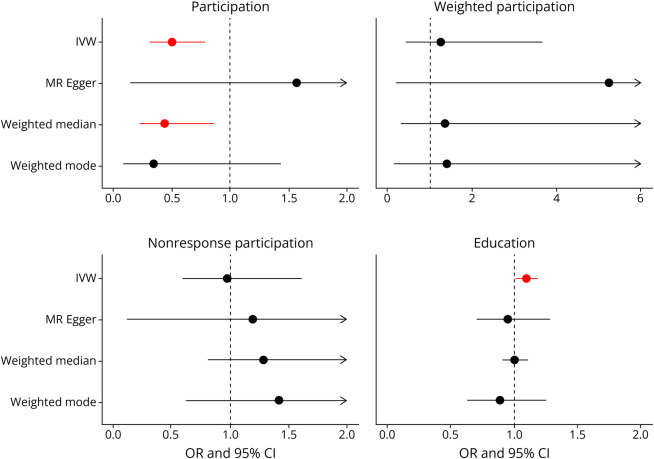
Univariable Odds Ratio of the Association Between Participation Measures, Education, and AD/ADRD Forest plots showing the MR odds ratio for the 4 different traits (participation, weighted participation, nonresponse participation, and education) and AD/ADRD based on the univariable analyses. Red represents significant results, and arrows represent CI intervals that continue past the x-axis. AD = Alzheimer disease.

The MR estimates suggested that an additional year of education reduced the odds of AD across all sensitivity analyses (OR_IVW_ 95% CI 0.70 [0.63–0.79], *p* = 8e-10; Table 1, Figure 3, and eFigure 2A), but our results had evidence of heterogeneity and pleiotropy. By contrast, the MR estimates suggested that an additional year of education increased the odds of AD/ADRD (OR_IVW_ 95% CI 1.09 [1.02–1.15], *p* = 0.006; Table 2, Figure 4, and eFigure 3A). There was evidence of heterogeneity (*p* = 2.39e-15), the MR Egger intercept was nonsignificant (*p* = 0.22), and the sensitivity analyses (OR_MR Egger_ 95% CI 0.912 [0.677–1.228], *p* = 0.54; OR_Weighted mode_95% CI 0.884 [0.635–1.23], *p* = 0.47) were consistent with the null.

**Table 1:** Summary of Univariable MR Results With AD

				Fixed-effects IVW		Random-effects IVW		MR Egger		WME		WMBE		Cochran Q test	MR Egger intercept
Exposure	Outcome	SNV	F Stats	Beta (se)	*p* Value	Beta (se)	*p* Value	Beta (se)	*p* Value	Beta (se)	*p* Value	Beta (se)	*p* Value	Q *p* value	*p* Value
Education	AD	464	55.1	−0.34 (0.06)	8e-10	−0.34 (0.06)	2.4e-08	−0.89 (0.23)	1.2e-04	−0.45 (0.08)	9.6e-08	−0.88 (0.31)	0.005	2e-04	0.01
Participation	AD	36	34.9	−1.12 (0.47)	0.01	−1.12 (0.47)	0.01	−1.04 (2.47)	0.67	−1.98 (0.62)	0.001	−3.11 (1.57)	0.05	0.10	0.97
Weighted participation	AD	18	31.3	−2.83 (1.1)	0.01	−2.83 (0.79)	0.0003	2.11 (3.22)	0.52	−3.24 (1.5)	0.03	−4.64 (2.53)	0.08	0.94	0.12
Nonresponse participation	AD	29	39.9	−0.45 (0.34)	0.18	−0.45 (0.34)	0.18	−1.28 (1.7)	0.46	−0.59 (0.4)	0.1	−1.44 (0.85)	0.1	0.033	0.02
Reverse causality															
AD	Education	27	105.2	0.004 (0.003)	0.22	0.004 (0.003)	0.22	0.007 (0.005)	0.22	0.01 (0.003)	0.002	0.009 (0.002)	0.003	1.71e-06	0.57
AD	Participation	28	107.3	−0.005 (0.001)	4.7e-05	−0.005 (0.001)	6.6e-04	−0.005 (0.002)	0.014	−0.002 (0.002)	0.19	−0.002 (0.002)	0.32	0.056	0.71
AD	Weighted participation	25	117.19	7.5e-05 (0.001)	0.95	7.5e-05 (0.001)	0.95	−3.9e-04 (0.001)	0.84	−6.14 (0.001)	0.97	1.83 (0.001)	0.99	0.28	0.73
AD	Nonresponse participation	24	117.19	−0.004 (0.002)	0.059	−0.004 (0.002)	0.10	−0.003 (0.003)	0.27	−0.005 (0.003)	0.11	−0.003 (0.003)	0.26	0.1	0.98

Abbreviations: AD = Alzheimer disease; IVW = inverse variance–weighted; WMBE = weighted mode–based estimator; WME = weighted median; SNV = single-nucleotide variation.

**Table 2:** Summary of Univariable MR Results With AD/ADRD

				Fixed-effects IVW		Random-effects IVW		MR Egger		WME		WMBE		Cochran Q test	MR Egger intercept
Exposure	Outcome	SNV	F Stats	Beta (se)	*p* Value	Beta (se)	*p* Value	Beta (se)	*p* Value	Beta (se)	*p* Value	Beta (se)	*p* Value	Q *p* value	*p* Value
Education	AD/ADRD	462	54.6	0.08 (0.03)	0.006	0.08 (0.04)	0.03	−0.09 (0.15)	0.54	0.0 (0.05)	1	−0.12 (0.17)	0.47	2.39e-15	0.22
Participation	AD/ADRD	37	34.9	−0.69 (0.23)	0.003	−0.69 (0.23)	0.003	0.44 (1.22)	0.71	−0.81 (0.34)	0.01	−1.07 (0.73)	0.15	0.46	0.34
Weighted participation	AD/ADRD	19	31.4	0.22 (0.54)	0.68	0.22 (0.47)	0.63	1.65 (1.7)	0.34	0.3 (0.77)	0.69	0.33 (1.26)	0.79	0.75	0.38
Nonresponse participation	AD/ADRD	30	39.6	0.02 (0.15)	0.87	0.02 (0.25)	0.92	−0.17 (1.21)	0.88	−0.25 (0.24)	0.3	−0.35 (0.47)	0.4	4e-07	0.87
Reverse causality															
AD/ADRD	Education	56	85.8	0.004 (0.005)	0.44	0.006 (0.004)	0.15	0.001 (0.009)	0.84	0.007 (0.004)	0.11	0.007 (0.004)	0.11	4.4e-18	0.77
AD/ADRD	Participation	55	85.3	−0.006 (0.001)	6.2e-05	−0.006 (0.002)	2.9e-03	−0.01 (0.003)	0.006	−0.006 (0.003)	0.04	−0.009 (0.004)	0.05	0.0003	0.19
AD/ADRD	Weighted participation	44	82.7	0.005 (0.001)	0.005	0.005 (0.001)	0.002	0.006 (0.003)	0.06	0.005 (0.002)	0.04	0.007 (0.003)	0.04	0.69	0.66
AD/ADRD	Nonresponse participation	49	78.8	−0.002 (0.003)	0.41	−0.002 (0.003)	0.48	−0.009 (0.006)	0.14	−0.006 (0.004)	0.16	−0.01 (0.005)	0.06	0.06	0.19

Abbreviations: AD = Alzheimer disease; IVW = inverse variance–weighted; SNV = single-nucleotide variation; WMBE = weighted mode–based estimator; WME = weighted median.

### Participation Is Associated With a Reduced Risk of AD

Participation in MHQ reduced the odds of AD (OR_IVW_ 95% CI 0.325 [0.128–0.326], *p* = 0.01; Table 1, Figure 3, and eFigure 2B), and this effect was supported by similar results in the weighted participation measure (OR_IVW_ 95% CI 0.0586 [0.0067–0.510], *p* = 0.01; Table 1, Figure 3, and eFigure 2C). MHQ decreased the odds of AD/ADRD (OR_IVW_ 95% CI 0.49 [0.31–0.79], *p* = 0.003; Table 2, Figure 4, and eFigure 3B).

### Bidirectional Analysis Showed an Effect of AD on Participation but No Effect of AD on Education

Genetic liability to AD reduced the odds of participation in the MHQ (OR_IVW_ 95% CI 0.994 [0.98–0.99], *p* = 4.7e-05); however, there was evidence of heterogeneity and potentially horizontal pleiotropy (Table 1 and eFigure 4B). Similarly, genetic liability to AD/ADRD reduced the odds of participation (OR_IVW_ 95% CI 0.993 [0.99–0.996], *p* = 6.24e-05; Table 2 and eFigure 5B), and the results were robust across the different sensitivity analyses. On the contrary, genetic liability to AD/ADRD increased the odds of weighted participation (OR_IVW_ 95% CI 1.005 [1.001–1.008], *p* = 0.005; Table 2 and eFigure 5C). There was limited evidence that genetic liability to AD or AD/ADRD affected educational attainment.

### Protective Effect of Education Remains After Accounting for Participation

**Figure 5:**
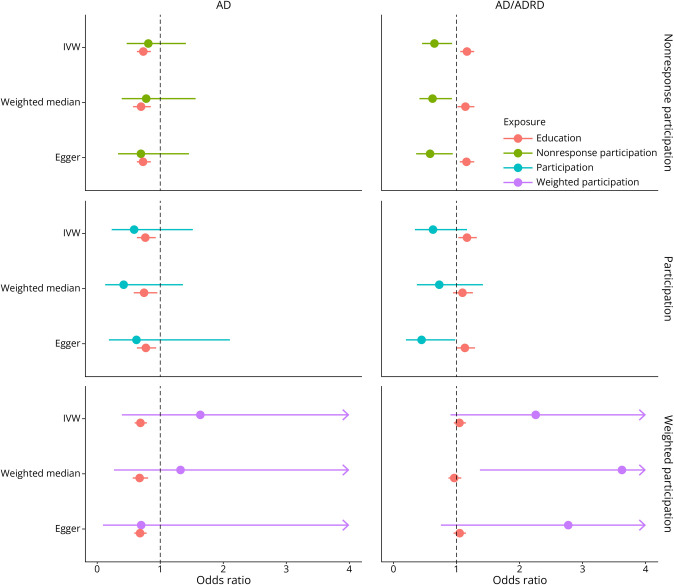
MVMR Odds Ratio of the Association Between Participation Measures and Education for AD and AD/ADRD Forest plot examining MVMR associations between education, 3 distinct participation measures, and clinical AD vs AD/ADRD. Each row represents a different participation measure, and arrows represent CI intervals that continue past the x-axis. AD = Alzheimer disease; MVMG = multivariable MR.

When adjusting for participation in the MHQ in MVMR analysis, an additional year of education continued to reduce the odds of AD (OR_IVW_ 95% CI 0.76 [0.62–0.92], *p* = 0.006; Figure 5 and eTable 2). Joint instrument strength was low for education and participation (conditional F-statistic for education = 3.41 and participation = 2.41; Figure 5 and eTable 2), suggestive of weak instruments for the multivariate analysis. After adjustment for education, there was limited evidence that participation affected AD (OR_IVW_ = 0.58, 95% CI [0.22–1.51], *p* = 0.27; Figure 5 and eTable 2) and this was supported by the weighted participation measure (OR_IVW_ = 1.63, 95% CI [0.39–6.69], *p* = 4.9e-01; Figure 5 and eTable 2). However, joint instrument strength was low for education and weighted participation (F-statistic for education = 10.86 and weighted participation = 1.42), which may result in weak instruments.

After adjusting for participation in the MHQ in MVMR, an additional year of education increased the odds of AD/ADRD (OR_IVW_ 95% CI 1.16 [1.03–1.32], *p* = 0.02; Figure 5 and eTable 3). However, there was significant heterogeneity, and joint instrument strength were weak (conditional F-statistic for education = 3.73 and participation = 2.48). This finding was supported when adjusting for nonresponse participation; years of education increased odds of AD/ADRD (OR_IVW_ 95% CI 1.16 [1.06–1.28], *p* = 0.002; Figure 5 and eTable 3) with robust findings, although instrument strength remained weak (conditional F-statistic for education = 7.42 and nonresponse participation = 2.79). We found limited evidence that weighted participation affected AD after adjustment for education (eTable 3).

The results from radial MR analysis are displayed in eFigures 6–9, and additional details about the methods can be found in eMethods. The SNPS used as IVs and their harmonized effects are listed in eTables 4–19.

## Discussion

This study used genetic correlations, univariable MR, and MVMR to evaluate the causal relationships between education, participation, and AD risk. Higher education was associated with a reduced risk of AD but an increased risk of AD/ADRD with evidence of heterogeneity, which could indicate horizontal pleiotropy, LD, or other mechanisms. Participation was also associated with a reduced risk of AD and AD/ADRD risk. Our multivariable analysis suggested that the estimated effect of education on AD was unlikely due to participation.

Education exhibited the strongest positive correlation with weighted participation, followed by participation and nonresponse participation. There were also significant negative correlations between educational attainment and AD, supporting consistent findings on education's protective effect on AD.^9,10^ Higher levels of education seem to play a crucial role in maintaining cognitive function across an extended lifespan.^34^ Educational attainment significantly contributes to cognitive reserve, an individual's ability to perform tasks and solve problems, even in the presence of amyloid pathology.^35^ Specifically, studies have shown that individuals with higher educational attainment exhibited better cognitive abilities among carriers of PSEN1 and E280A (mutations predisposing individuals to early-onset AD).^36^ Education is thought to contribute to cognitive reserve by increasing synaptic density in the neocortical association cortex.^37^

Across both AD and AD/ADRD data sets, participation in the MHQ was associated with a reduced risk of AD. This finding was supported by a significant negative genetic correlation between participation and AD. Factors influencing participation in clinical research include lower socioeconomic status (lower wages, lower quality neighborhoods, higher unemployment, and household overcrowding) and lower educational achievement, which are risk factors of AD and are closely linked to the risk of cognitive impairment.^38,39^ Participation bias in studies could lead to nonrepresentative samples, which can produce results that are not generalizable to other populations.^11,13,17^

Participation and selection bias can be a form of collider bias because they can induce a spurious association between the exposure and outcome that may not exist in the general population. To address this, we compared different participation measures, including the weighted participation measure. This model predicts UK Biobank participation based on auxiliary variables related to health, lifestyle, and demographics, capturing a dimension of participation different from the optional MHQ. In addition, the weighted participation measure uses IPW to correct for selection biases by weighting individuals who are underrepresented (low probability of participation) and downweighing those who are overrepresented (high probability of participation). Because the weighted participation estimates showed little change, we conclude that participation in the MHQ did not act as a collider or distort the original MVMR estimates. However, unmeasured confounding remains a possibility in the MVMR framework (eTable 2).

The bidirectional analysis in both the AD and AD/ADRD data sets provided evidence supporting an apparent causal effect of genetic liability to AD on participation. The analysis suggests that AD pathogenesis may influence participation in cohort studies in midlife because cognitive deficits can be detectable up to 10–12 years before clinical diagnosis of AD dementia.^40^ This aligns up with the development of AD pathology at least 15 years before diagnosis.^41^ Genetic liability to AD/ADRD was associated with a small decrease in participation but a small increase in weighted participation. This discrepancy in the weighted participation measure analysis can be attributed to the use of proxy measures for both AD and participation, which may lead to inaccuracies. Nevertheless, the effect of AD on participation can largely be explained through education; lower educational levels are linked to a decreased likelihood of participation among individuals with AD.^42^

To distinguish the individual impacts of participation and education on AD, we conducted MVMR. Our MVMR analysis, adjusting for participation, resulted in similar estimates of the effect of education on AD. These results suggest that the effect of years of education on AD found using MR studies is unlikely to be due to participation bias but is likely mediated by other mechanisms. Given the mounting evidence supporting the effect of education on risk of AD, it becomes crucial to analyze public policies encouraging sustained education and to discover the molecular, familial, and societal mechanisms that mediate these effects.^43^ Research indicates that the reduction in the incidence of dementia may be due to educational policy.^44^

Discrepancies in results emerged between the AD and AD/ADRD data sets. Notably, the univariable MR analysis indicated that education had a protective effect on AD but a risk-increasing effect for AD/ADRD. This effect is likely due to survival bias and reporting bias.^45^ The AD/ADRD cases in the Bellenguez data set largely comprise individuals who reported parental dementia, and individuals reporting parental AD diagnoses may have parents with less genetic risk of other diseases, such as cardiovascular disease and cancer, allowing them to live longer. More educated parents will be older when they have kids and will have more time to develop AD, which is supported by positive genetic correlations between age at first birth and educational attainment.^46^ In addition, only individuals aware of their parent's health typically report parental AD diagnoses.^45^ While efforts can be made to mitigate AD biases in GWAX, such as controlling for parental age and vital status, the ultimate solution lies in enhancing the quality of GWAX analysis. These discrepancies highlight the importance of using GWAS data sets containing clinical or neuropathologic defined cases over family history-based proxy phenotypes for MR studies.^47^ However, the small sample sizes of many GWAS data sets can pose challenges for epidemiologic research.

Strengths of our study include using complementary measures of participation, which allowed us to see the effectiveness of different methods; a bidirectional MR approach to confirm the direction of causality; comparison of clinical vs proxy data sets; and multiple sensitivity analyses to confirm the robustness of the results. Nevertheless, our study findings come with several limitations. First, while F-statistics for the univariable analyses exceed the standard threshold of 10, the conditional F-statistics in our MVMR analyses were below 10. This indicates that causal estimates in the MVMR analyses may be affected by weak instrument bias. Furthermore, the F-statistics for educational attainment were generally larger than those of the participation data sets, resulting in more precise estimates for education than participation. There has been a lack of highly powered participation data sets, and future work can focus on implementing methods to address weak instrument bias.^48^ Second, the data sets exclusively include individuals of European ancestry, limiting the generalizability of our results to the broader population. Third, biases might arise when there is a proportion of sample overlap between data sets.^49^ Last, the associations between participation and AD could be driven by confounding, as participation in the MHQ and other participation measures have been linked to demographic and behavioral factors, such as depression, which are themselves associated with AD.^50^

In conclusion, we found that the MR estimates of the effect of years of education on AD are unlikely to be due to participation bias. Education increased the odds of AD/ADRD in our GWAX data set, likely attributed to survival bias and reporting bias, highlighting the importance of using clinical case-control AD GWASs in MR analyses. These findings contribute to the accumulating evidence supporting the protective role of education against AD. We urgently need to discover the mechanisms that explain these effects and develop effective interventions to reduce the incidence of AD in the population.

## Glossary

AD: Alzheimer disease
GWAS: genome-wide association study
GWAX: GWAS by proxy
HRS: Health and Retirement Study
IPW: inverse probability weighting
IVW: inverse variance–weighted
LD: linkage disequilibrium
MHQ: Mental Health Questionnaire
MR: Mendelian randomization
MVMR: multivariable MR
NACC: National Alzheimer's Coordinating Center
SNV: single-nucleotide variation
WMBE: weighted mode–based estimator
WME: weighted median

## References

[1] Farfel JM, Nitrini R, Suemoto CK, et al. Very low levels of education and cognitive reserve: a clinicopathologic study. Neurology. 2013;81(7):650-657. doi:10.1212/WNL.0b013e3182a08f1b 23873971 PMC3775692

[2] Manton KC, Gu XL, Ukraintseva SV. Declining prevalence of dementia in the U.S. elderly population. Adv Gerontol Uspekhi Gerontol. 2005;16:30-37. 16075674

[3] Spitzer S. Biases in health expectancies due to educational differences in survey participation of older Europeans: it's worth weighting for. Eur J Health Econ. 2020;21(4):573-605. doi:10.1007/s10198-019-01152-0 31989388 PMC7214500

[4] Scanlon JK, Wofford L, Fair A, Philippi D. Predictors of participation in clinical research. Nurs Res. 2021;70(4):289-297. doi:10.1097/NNR.0000000000000513 33883501 PMC8231664

[5] Arce Rentería M, Mobley TM, Evangelista ND, et al. Representativeness of samples enrolled in Alzheimer's disease research centers. Alzheimer's Dement. 2023;15(2):e12450. doi:10.1002/dad2.12450 PMC10242202 37287650

[6] Munafò MR, Tilling K, Taylor AE, Evans DM, Davey Smith G. Collider scope: when selection bias can substantially influence observed associations. Int J Epidemiol. 2018;47(1):226-235. doi:10.1093/ije/dyx206 29040562 PMC5837306

[7] Davies NM, Holmes MV, Davey Smith G. Reading Mendelian randomisation studies: a guide, glossary, and checklist for clinicians. BMJ 2018;362:k601. doi:10.1136/bmj.k601 30002074 PMC6041728

[8] Sanderson E, Glymour MM, Holmes MV, et al. Mendelian randomization. Nat Rev Methods Primers. 2022;2(1):6-21. doi:10.1038/s43586-021-00092-5 37325194 PMC7614635

[9] Anderson EL, Howe LD, Wade KH, et al. Education, intelligence and Alzheimer's disease: evidence from a multivariable two-sample Mendelian randomization study. Int J Epidemiol. 2020;49(4):1163-1172. doi:10.1093/ije/dyz280 32003800 PMC7660137

[10] Seyedsalehi A, Warrier V, Bethlehem RAI, Perry BI, Burgess S, Murray GK. Educational attainment, structural brain reserve and Alzheimer's disease: a Mendelian randomization analysis. Brain. 2023;146(5):2059-2074. doi:10.1093/brain/awac392 36310536 PMC10151197

[11] Tyrrell J, Zheng J, Beaumont R, et al. Genetic predictors of participation in optional components of UK Biobank. Nat Commun. 2021;12(1):886. doi:10.1038/s41467-021-21073-y 33563987 PMC7873270

[12] van Alten S, Domingue BW, Faul J, Galama T, Marees AT. Reweighting UK Biobank corrects for pervasive selection bias due to volunteering. Int J Epidemiol. 2024;53(3):dyae054. doi:10.1093/ije/dyae054 38715336 PMC11076923

[13] Schoeler T, Speed D, Porcu E, Pirastu N, Pingault JB, Kutalik Z. Participation bias in the UK Biobank distorts genetic associations and downstream analyses. Nat Hum Behav. 2023;7(7):1216-1227. doi:10.1038/s41562-023-01579-9 37106081 PMC10365993

[14] Gkatzionis A, Burgess S. Contextualizing selection bias in Mendelian randomization: how bad is it likely to be? Int J Epidemiol. 2019;48(3):691-701. doi:10.1093/ije/dyy202 30325422 PMC6659463

[15] Carter AR, Sanderson E, Hammerton G, et al. Mendelian randomisation for mediation analysis: current methods and challenges for implementation. Eur J Epidemiol. 2021;36(5):465-478. doi:10.1007/s10654-021-00757-1 33961203 PMC8159796

[16] Okbay A, Wu Y, Wang N, et al. Polygenic prediction of educational attainment within and between families from genome-wide association analyses in 3 million individuals. Nat Genet. 2022;54(4):437-449. doi:10.1038/s41588-022-01016-z 35361970 PMC9005349

[17] Mignogna G, Carey CE, Wedow R, et al. Patterns of item nonresponse behaviour to survey questionnaires are systematic and associated with genetic loci. Nat Hum Behav. 2023;7(8):1371-1387. doi:10.1038/s41562-023-01632-7 37386106 PMC10444625

[18] Kunkle BW, Grenier-Boley B, Sims R, et al. Genetic meta-analysis of diagnosed Alzheimer's disease identifies new risk loci and implicates Aβ, tau, immunity and lipid processing. Nat Genet. 2019;51(3):414-430. doi:10.1038/s41588-019-0358-2 30820047 PMC6463297

[19] Bellenguez C, Küçükali F, Jansen IE, et al. New insights into the genetic etiology of Alzheimer's disease and related dementias. Nat Genet. 2022;54(4):412-436. doi:10.1038/s41588-022-01024-z 35379992 PMC9005347

[20] Murphy AE, Schilder BM, Skene NG. MungeSumstats: a Bioconductor package for the standardization and quality control of many GWAS summary statistics. Bioinformatics. 2021;37(23):4593-4596. doi:10.1093/bioinformatics/btab665 34601555 PMC8652100

[21] Bulik-Sullivan B, Finucane HK, Anttila V, et al. An atlas of genetic correlations across human diseases and traits. Nat Genet. 2015;47(11):1236-1241. doi:10.1038/ng.3406 26414676 PMC4797329

[22] van Rheenen W, Peyrot WJ, Schork AJ, Lee SH, Wray NR. Genetic correlations of polygenic disease traits: from theory to practice. Nat Rev Genet. 2019;20(10):567-581. doi:10.1038/s41576-019-0137-z 31171865

[23] Myers TA, Chanock SJ, Machiela MJ. LDlinkR: an R package for rapidly calculating linkage disequilibrium statistics in diverse populations. Front Genet. 2020;11:157. doi:10.3389/fgene.2020.00157 32180801 PMC7059597

[24] Hemani G, Zheng J, Elsworth B, et al. The MR-Base platform supports systematic causal inference across the human phenome. eLife. 2018;7:e34408. doi:10.7554/eLife.34408 29846171 PMC5976434

[25] Hemani G, Tilling K, Davey Smith G. Orienting the causal relationship between imprecisely measured traits using GWAS summary data. PLoS Genet. 2017;13(11):e1007081. doi:10.1371/journal.pgen.1007081 29149188 PMC5711033

[26] PhenoScanner. Updated 2019. Accessed January 18, 2024. phenoscanner.medschl.cam.ac.uk/

[27] Hemani G, Bowden J, Davey Smith G. Evaluating the potential role of pleiotropy in Mendelian randomization studies. Hum Mol Genet. 2018;27(R2):R195-R208. doi:10.1093/hmg/ddy163 29771313 PMC6061876

[28] Bowden J, Spiller W, Del Greco M F, et al. Improving the visualization, interpretation and analysis of two-sample summary data Mendelian randomization via the Radial plot and Radial regression. Int J Epidemiol. 2018;47(4):1264-1278. doi:10.1093/ije/dyy101 29961852 PMC6124632

[29] Bowden J, Davey Smith G, Burgess S. Mendelian randomization with invalid instruments: effect estimation and bias detection through Egger regression. Int J Epidemiol. 2015;44(2):512-525. doi:10.1093/ije/dyv080 26050253 PMC4469799

[30] Bowden J, Davey Smith G, Haycock PC, Burgess S. Consistent estimation in mendelian randomization with some invalid instruments using a weighted median estimator. Genet Epidemiol. 2016;40(4):304-314. doi:10.1002/gepi.21965 27061298 PMC4849733

[31] Hartwig FP, Davey Smith G, Bowden J. Robust inference in summary data Mendelian randomization via the zero modal pleiotropy assumption. Int J Epidemiol. 2017;46(6):1985-1998. doi:10.1093/ije/dyx102 29040600 PMC5837715

[32] Sanderson E, Spiller W, Bowden J. Testing and correcting for weak and pleiotropic instruments in two-sample multivariable Mendelian randomization. Stat Med. 2021;40(25):5434-5452. doi:10.1002/sim.9133 34338327 PMC9479726

[33] MendelianRandomization v0. Wellcome Open Research. 2023;8:449. Accessed April 1, 2024. wellcomeopenresearch.org/articles/8-449/v2

[34] Amieva H, Mokri H, Le Goff M, et al. Compensatory mechanisms in higher-educated subjects with Alzheimer's disease: a study of 20 years of cognitive decline. Brain. 2014;137(Pt 4):1167-1175. doi:10.1093/brain/awu035 24578544

[35] Zhu W, Li X, Li X, et al. The protective impact of education on brain structure and function in Alzheimer's disease. BMC Neurol. 2021;21(1):423. doi:10.1186/s12883-021-02445-9 34717581 PMC8557004

[36] Langella S, Barksdale NG, Vasquez D, et al. Effect of apolipoprotein genotype and educational attainment on cognitive function in autosomal dominant Alzheimer's disease. Nat Commun. 2023;14(1):5120. doi:10.1038/s41467-023-40775-z 37612284 PMC10447560

[37] Valenzuela MJ. Cognitive Reserve in the Aging Brain. In: Oxford Research Encyclopedia of Psychology; 2019. doi:10.1093/acrefore/9780190236557.013.338

[38] Pandya N. Factors influencing participation in clinical research: do minority and lower socioeconomic status patients experience greater barriers in participation? Res Sch Poster Present. 2014;2-4. //scholarlyworks.lvhn.org/research-scholars-posters/357

[39] Lower Socioeconomic Status Linked to Dementia | AAIC | alz.Org. AAIC. 2022. Accessed November 22, 2023. //aaic.alz.org/releases_2022/lower-socioeconomic-dementia-risk.asp

[40] Caselli RJ, Langlais BT, Dueck AC, et al. Neuropsychological decline up to 20 years before incident mild cognitive impairment. Alzheimer's Dement. 2020;16(3):512-523. doi:10.1016/j.jalz.2019.09.085 31787561 PMC7067658

[41] Aisen PS, Cummings J, Jack CR, et al. On the path to 2025: understanding the Alzheimer's disease continuum. Alzheimers Res Ther. 2017;9(1):60. doi:10.1186/s13195-017-0283-5 28793924 PMC5549378

[42] Reinikainen J, Tolonen H, Borodulin K, et al. Participation rates by educational levels have diverged during 25 years in Finnish health examination surveys. Eur J Public Health. 2018;28(2):237-243. doi:10.1093/eurpub/ckx151 29036286

[43] Barcellos S, Carvalho L, Langa K, Nimmagadda S, Turley P. Education and Dementia Risk. National Bureau of Economic Research; 2025:w33430. doi:10.3386/w33430

[44] Mukadam N, Wolters FJ, Walsh S, et al. Changes in prevalence and incidence of dementia and risk factors for dementia: an analysis from cohort studies. Lancet Public Health. 2024;9(7):e443-e460. doi:10.1016/S2468-2667(24)00120-8 38942556

[45] Wu Y, Sun Z, Zheng Q, et al. Pervasive biases in proxy GWAS based on parental history of Alzheimer's disease. bioRxiv. 2023:2023.10.13.562272. doi:10.1101/2023.10.13.562272 PMC11929606 39496879

[46] Mills MC, Tropf FC, Brazel DM, et al. Identification of 371 genetic variants for age at first sex and birth linked to externalising behaviour. Nat Hum Behav. 2021;5(12):1717-1730. doi:10.1038/s41562-021-01135-3 34211149 PMC7612120

[47] Escott-Price V, Hardy J. Genome-wide association studies for Alzheimer's disease: bigger is not always better. Brain Commun. 2022;4(3):fcac125. doi:10.1093/braincomms/fcac125 35663382 PMC9155614

[48] Grant AJ, Burgess S. A Bayesian approach to Mendelian randomization using summary statistics in the univariable and multivariable settings with correlated pleiotropy. Am J Hum Genet. 2024;111(1):165-180. doi:10.1016/j.ajhg.2023.12.002 38181732 PMC10806746

[49] Burgess S, Davies NM, Thompson SG. Bias due to participant overlap in two‐sample Mendelian randomization. Genet Epidemiol. 2016;40(7):597-608. doi:10.1002/gepi.21998 27625185 PMC5082560

[50] Caraci F, Copani A, Nicoletti F, Drago F. Depression and Alzheimer's disease: neurobiological links and common pharmacological targets. Eur J Pharmacol. 2010;626(1):64-71. doi:10.1016/j.ejphar.2009.10.022 19837057

